# Stromal Filters in Automated Immunostain Scoring

**DOI:** 10.1155/2014/497426

**Published:** 2014-12-30

**Authors:** Kunal Patel, Anthony Bui, Greg Riedlinger, Yukako Yagi

**Affiliations:** ^1^Massachusetts General Hospital, 55 Fruit Street, Boston, MA 02114, USA; ^2^Boston Children's Hospital, 300 Longwood Avenue, Boston, MA 02115, USA

## Introduction

KI-67 is a marker for cell proliferation which binds a nuclear antigen and is thus a prime antibody in immunohistochemistry. Scoring methodologies derived from KI-67 immunostaining have shown promise as predictors of lethality in a range of cancers [1]. The most common scoring method is the labelling index, a ratio of KI-67 positive cells to the entire population [1].

Manual calculation of a labelling index can be a time-consuming process for pathologists, who count cells in a bright field. Automated scoring algorithms and programs have been written to generate labelling indices, but they are nonspecific with respect to cell type and most often skew results by including stromal cells in the index. This problem is particularly relevant in breast cancer, where tumors are often located in a field of fibroadipose tissue. We have adapted algorithms for immunostain scoring and tested 2 stromal cell filtration algorithms which remove these cells based on their elongated nuclear morphology.

## Methods

An automated scoring algorithm was adapted from ImmunoRatio [2] and written in Python programming language. Within this scoring program, two methods of stromal filtration were tested. The first filtered cells below a threshold defined by the isoperimetric quotient of labeled cells, while the second used a shape property known as the Hu moment invariant. The algorithm was applied to a dataset of breast cancer images, and results were correlated with nonstromal filtration and a pathologist score.

## Results

Preliminary results show that algorithms with stromal filtration correlate better with pathology results than nonfiltered counterparts. Additionally, the Hu moment invariant is a better variable for stromal cell filtration due to its nonreliance on perimeter. Deviation of automatic scores from pathologist-scored results is directly correlated with the amount of stromal tissue in the image field.

## Conclusions

Stromal cell filtration is a promising technique in the development of automatic scoring algorithms. Additional methods should be explored to address the filtration of nonelongated stromal nuclei. With improvements in usability, it can be integrated into whole slide imaging systems in the near future.
